# A Rare Tumor in the Common Bile Duct: A Case Report

**DOI:** 10.1089/pancan.2016.0020

**Published:** 2017-02-01

**Authors:** Mustafa Suker, Katharina Biermann, Casper van Eijck, Michael Doukas

**Affiliations:** ^1^ Department of Surgery, Erasmus MC, University Medical Center Rotterdam, Rotterdam, The Netherlands.; ^2^ Department of Pathology, Erasmus MC, University Medical Center Rotterdam, Rotterdam, The Netherlands.

**Keywords:** biliary tract neoplasms, Epstein–Barr virus infections, lymphoepithelial-like carcinoma

## Abstract

**Background:** Lymphoepithelial-like carcinoma (LEC) is rarely found in organs outside the nasopharyngeal area. This is the first case report of Epstein–Barr virus (EBV)-associated LEC of the extrahepatic tract. As it is very difficult to distinguish between LEC and adenocarcinoma in the clinical presentation, this article can give more insight into how the pathological analysis can help with the diagnosis.

**Case presentation:** A 37-year-old Caucasian male with a history of Crohn's disease and primary sclerosing cholangitis presented with cholestasis. A computed tomography scan revealed a tumor in the pancreatic head without invasion into the surrounding organs. The patient underwent an uncomplicated pylorus-preserving pancreaticoduodenectomy, with pathology revealing an epithelial carcinoma of the common bile duct with metastases in 4 of the 18 resected lymph nodes. *In situ* hybridization demonstrated extensive EBV positivity in the tumor cells, and in serum, positive IgG anti-EBV was found. The diagnosis of EBV-associated LEC was hereby confirmed. The postoperative course was uneventful and 18 months after surgery there is no recurrence.

**Conclusion:** In the case of an epithelial tumor in the periampullary region, one should consider EBV-associated LEC as this tumor may have a lot of similarity with the adenocarcinoma but has lower rates of recurrence after surgery and better overall survival.

## Introduction and Background

Lymphoepithelial-like carcinoma (LEC) is usually seen in nasopharyngeal carcinoma and mostly associated with Epstein–Barr virus (EBV) infection.^1^ However, case reports have described the occurrence of LEC in the lung, salivary gland, trachea, thymus, esophagus, urinary bladder, uterine cervix, vagina, breast, renal pelvis, stomach, and the intrahepatic biliary tract. The diagnosis of biliary tract LEC is of importance to the physician as well to the patient, as these tumors are characterized by lower rates of recurrence and better overall survival.^2^ Here, we report the first EBV-related LEC of the extrahepatic biliary tract.

## Case Presentation

**FIG. 1.**
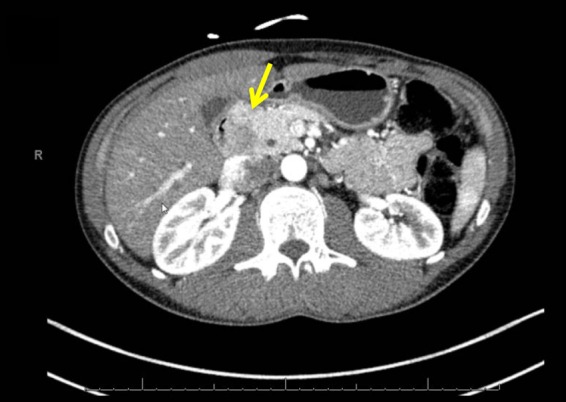
Computed tomography scan with the yellow arrow pointing at the tumor.

A 37-year-old Caucasian male with a history of Crohn's disease and primary sclerosing cholangitis presented with cholestasis. A magnetic resonance cholangiopancreatography showed a tumorous process in the pancreatic head with a double duct sign. Endoscopic ultrasound revealed a hypoechoic lesion of 3 cm in the pancreatic head with a pancreatic duct dilatation of 5.5 mm. Endoscopic retrograde cholangiopancreatogram was performed with placement of a pigtail stent in the pancreatic duct, but cannulation of the common bile duct was not possible. A computed tomography scan revealed a tumor in the pancreatic head without invasion into the surrounding organs (Fig. 1).

**FIG. 2.**
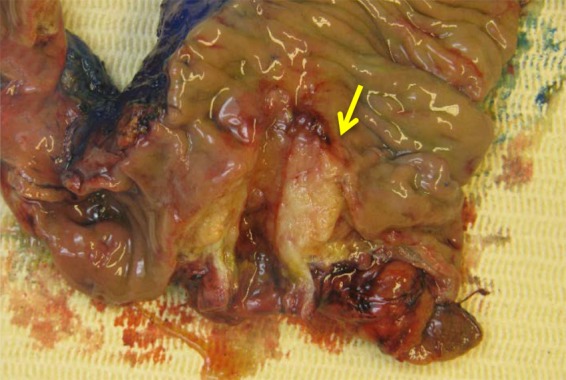
Resection specimen with the yellow arrow pointing to the tumor in the common bile duct.

**FIG. 3.**
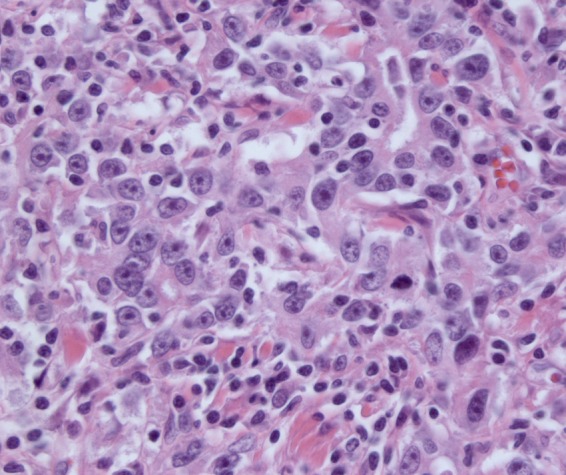
Poorly differentiated carcinoma of the common bile duct on hematoxylin and eosin stain × 400.

**FIG. 4.**
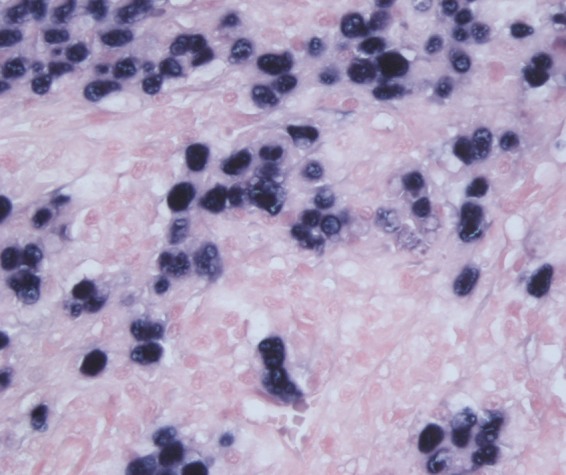
*In situ* hybridization demonstrating extensive EBV positivity in the tumor cells on EBV-encoded RNA stain × 400. EBV, Epstein–Barr virus.

As our working diagnosis was pancreatic cancer, our patient underwent an uncomplicated pylorus-preserving pancreaticoduodenectomy. The surgical specimen revealed a tumor arising in the common bile duct with pancreatic involvement, and possible extension to the duodenum and into surrounding fat tissue (Fig. 2). The histopathological sections showed poorly differentiated carcinoma of the common bile duct mostly with syncytial growth and prominent lymphoplasmacellular inflammatory component, consistent with LEC. Immunohistochemistry confirmed epithelial origin, since all tumor cells were positive for keratins (pankeratin and CAM5.2) and keratin-19. *In situ* hybridization demonstrated extensive EBV positivity in the tumor cells (Fig. 3: hematoxylin and eosin stain × 400 and Fig. 4: EBV-encoded RNA stain × 400). Eighteen specimen lymph nodes were identified and four lymph nodes revealed carcinoma metastases with histopathological features of the primary. In the serum, IgG anti-EBV was positive while IgM anti-EBV was negative. The diagnosis of EBV-related LEC was confirmed by professor R.H. Hruban (Johns Hopkins Hospital, Baltimore, USA). Postoperative course was uneventful for our patient and 18 months after surgery there is no carcinoma recurrence.

## Conclusion

EBV-associated LEC is rarely found in organs outside the nasopharyngeal area. Case reports described the occurrence of LEC in virtually every organ.^2^ To the best of our knowledge, there is only one published case that reported LEC in the distal bile duct,^3^ our case being the first EBV-related carcinoma of the extrahepatic biliary tract.

## Abbreviations Used

EBV: Epstein–Barr virus
LEC: lymphoepithelial-like carcinoma
